# EmbRS a new two-component system that inhibits biofilm formation and saves *Rubrivivax gelatinosus* from sinking

**DOI:** 10.1002/mbo3.82

**Published:** 2013-03-21

**Authors:** Anne Soisig Steunou, Sylviane Liotenberg, Marie-Noêlle Soler, Romain Briandet, Valérie Barbe, Chantal Astier, Soufian Ouchane

**Affiliations:** 1 CNRS, CGM, UPR 3404, Université Paris Sud1 Ave. de la Terrasse, Gif-sur-Yvette, F-91198, France; 2 CNRS, Plate forme de Microscopie et de Biologie Cellulaire, IFR87/Imagif, (FRC3115)F-91198, Gif-sur-Yvette, France; 3 INRA, AgroParisTech, UMR 1319 MICALISF-78350, Jouy-en-Josas, France; 4 CEA, DSV, IG,GenoscopeF-91057, Evry Cedex, France

**Keywords:** Biofilm formation, biofilm regulation, burkholderiales, exopolysaccharides, phototrophic biofilm, two-component system

## Abstract

Photosynthetic bacteria can switch from planktonic lifestyle to phototrophic biofilm in mats in response to environmental changes. The mechanisms of phototrophic biofilm formation are, however, not characterized. Herein, we report a two-component system EmbRS that controls the biofilm formation in a photosynthetic member of the Burkholderiales order, the purple bacterium *Rubrivivax gelatinosus*. EmbRS inactivation results in cells that form conspicuous bacterial veils and fast-sinking aggregates in liquid. Biofilm analyses indicated that EmbRS represses the production of an extracellular matrix and biofilm formation. Mapping of transposon mutants that partially or completely restore the wild-type (WT) phenotype allowed the identification of two gene clusters involved in polysaccharide synthesis, one fully conserved only in *Thauera sp*., a floc-forming wastewater bacterium. A second two-component system BmfRS and a putative diguanylate cyclase BdcA were also identified in this screen suggesting their involvement in biofilm formation in this bacterium. The role of polysaccharides in sinking of microorganisms and organic matter, as well as the importance and the evolution of such regulatory system in phototrophic microorganisms are discussed.

## Introduction

Photosynthetic bacteria are versatile microorganisms that can grow either as plankton or aggregates in phototrophic biofilms and colonize diverse aquatic environments. Phototrophic biofilms, referred to as microbial phototrophic mats, usually grow on surfaces when exposed to light. These mats can contain diatoms, algae, cyanobacteria, purple and green bacteria and even nonphototrophic microorganisms (De Philippis et al. 2005; Roeselers et al. 2008). In these structures, microorganisms produce extracellular polymeric substances that form the matrix of the biofilm. In spite of the potential interest in the application of phototrophic biofilms, the economic and health impact resulting from the development of phototrophic biofilms, the mechanisms and regulatory pathways leading to the formation of the microbial phototrophic biofilms remain under investigated.

Central to sensing and responding to environmental signals in bacteria are the two-component systems. Examples of two-component systems that respond to a wide range of signals have been reported in the literature and are reviewed in depth in recent reviews (Gao and Stock 2009; Krell et al. 2010; Wuichet and Zhulin 2010). However, the precise physiological functions of many putative two-component systems remain still unknown. Genetic analyses of signal transduction proteins and cascades may lead to new biological discoveries and unveil new regulatory circuitry.

Many two-component systems, in particular those involved in the regulation of photosynthesis in response to light, oxygen, and nitrogen availability, have been characterized in phototrophic bacteria (Bauer et al. 2003; Zeilstra-Ryalls and Kaplan 2004; Gomelsky et al. 2008; Hassani et al. 2010; Gomelsky and Hoff 2011). However, regulatory pathways involved in the transition from planktonic to biofilm lifestyles have not yet been identified. *Rubrivivax gelatinosus* is a purple nonsulfur, burkholderiale, β-proteobacterium that grows by photosynthesis or aerobic respiration. The photosynthetic gene cluster (PGC) encoding most of the genes involved in photosynthesis in this bacterium has been previously described (Nagashima et al. 1994; Liotenberg et al. 2008). Two genes encoding a putative two-component system were identified in the vicinity of the PGC cluster. Their localization 276 nucleotides downstream *bchP* in the PGC suggested that this system may be involved in regulation of photosynthesis in *Rubrivivax*.

To examine the role of this two-component system (henceforth named *embRS* for extracellular matrix and biofilm) in photosynthesis, the *embR* and *embS* genes were inactivated. The analyses of the *embS* or *embR* disrupted strains indicate that *embRS* is not directly required for photosynthesis. Intriguingly, the inactivation of *embR or embS* revealed an interesting mode of growth in purple bacteria. The mutants presented rugose and rough colony morphologies and when grown in liquid medium, these mutations led to the autoaggregation and fast sinking of the cells in a compact mass. Transposon mutagenesis was undertaken to uncover the genes responsible for this phenotype and potentially to identify the targets and partners of the EmbRS system. The results provide new insight into the molecular and regulatory mechanisms by which *Rubrivivax* switches between planktonic and biofilm lifestyles and perhaps unravel an evolutionary step toward photosynthetic lifestyle of this bacterium.

## Experimental Procedures

### Bacterial strains and media

*Escherichia coli* was grown at 37°C on LB medium. *R. gelatinosus* was grown at 30°C, in the dark semiaerobically or in light anaerobically (photosynthetic: PS) in filled and sealed tubes, in malate medium. Bacterial strains and plasmids are listed in Table S1. Transformation of *R. gelatinosus* was carried out by electroporation as described in (Ouchane et al. 1996). Antibiotics were used at the following concentrations: Kanamycin (km), Streptomycin/Spectinomycin (Ω), and Trimethoprim (Tp) were at 50 μg/mL and tetracycline 2 μg/mL.

### Gene transfer and strain selection

Transformation of *R. gelatinosus* cells was performed by electroporation (Ouchane et al. 1996). Transformants were selected on malate plates supplemented with the appropriate antibiotic under aerobic conditions. Following transformant selection, template genomic DNA was prepared from the ampicillin-sensitive transformants (double crossover) and confirmation of the antibiotic resistance marker's presence at the desired locus was performed by polymerase chain reaction (PCR).

### Transposon mutagenesis and mutant selection

*Rubrivivax gelatinosus* ΔEmbRS mutant was mutagenized using the EZ-Tn5 Transposome Kit (Epicentre, Madison, WI, USA) by following the manufacturer's protocol and as described in (Ouchane et al. 1996) for electroporation.

### Transposon mapping and cloning of mutated genes

DNA was prepared from the isolated mutants. Approximately, 2 μg of DNA was digested with PstI, ligated with PstI linearized KS bluescript, and transformed into *E. coli*. Transformants were selected on LB agar plates containing kanamycin. Plasmids were isolated and DNA flanking the Tn5 was sequenced with Tn5 Rev primer supplied with EZ::Tn5 kit.

### Gene cloning and plasmid constructions for allele replacement

Standard methods were performed according to (Sambrook et al. 1989) unless indicated otherwise. The sequences reported in this article were deposited in Gene Bank with accession number: FO082878: *psc2*. FO082879: *psc1*. FO082880: *RGS1_10841*. FO082881: *RGS1_30021*. FO082882: *RGS1_30022*. FO082883: *RGS1_40251*. FO0828-84: RGS1_80151. FO082885: *RGS1_80321*. FO082886: *RG-S1_80322*.

**Figure 1:**
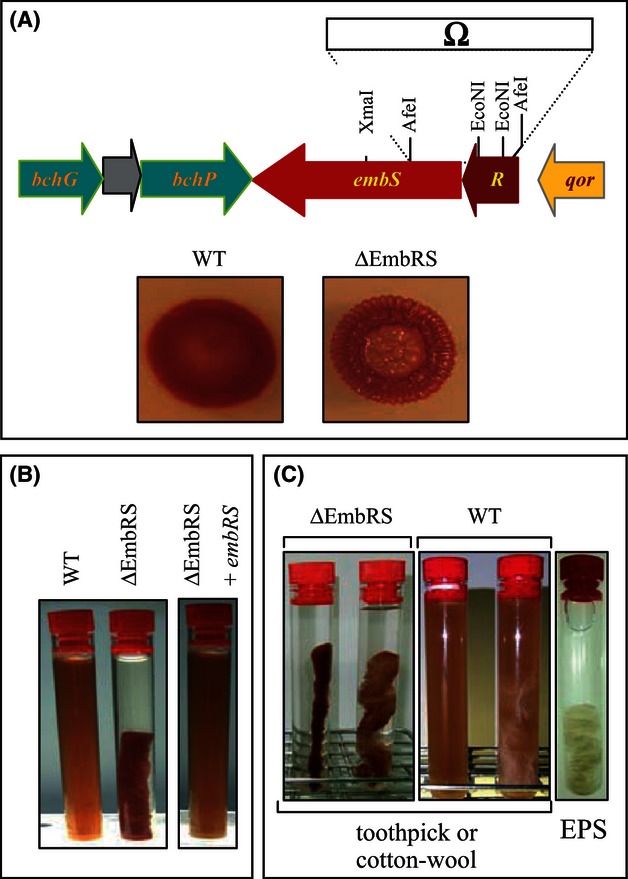
Genetic organization of the *ebmRS* operon and the phenotype of the corresponding mutant compared to the WT. (A) The flanking regions and the restriction sites used to generate *embRS* mutants are shown. (B) PS growth phenotype in liquid of the ΔEmbRS mutant and the complemented strain (ΔEmbRS + *embRS*). (C) Adherence and attachment abilities of the ΔEmbRS mutant to solid substrates (toothpick or cotton wool). EPS: The extracellular polymeric substance that encased the cells was isolated upon cell removal.

To clone the *embS* gene from genomic DNA of *R. gelatinosus*, a 2.15-kb fragment was amplified by PCR using the primers ol-468 and ol-459 (Table S2). The fragment was cloned into the PCR cloning vector pGEM-T to give pSMS. The *embS* gene was inactivated by the insertion of the Ω cassette at the unique XmaI site (Fig. 1A) within the *embS* coding sequence. Briefly, plasmid pSMS was subjected to restriction enzyme digestion and treated with T4 polymerase, prior to ligation with the SmaI-digested Ω cassette (2 kb). The resulting recombinant plasmids was designated pSMSΩ.

A 2.6-kb *embRS* fragment was cloned into pGEM-T using the primers ol-489 and ol-475 to give pSMSR. *embR* gene was deleted by the insertion of the Km cassette at the EcoNI sites (Fig. 1A) within the *embR* coding sequence. To that aim, pSMSR was subjected to restriction enzyme digestion and treated with T4 polymerase, prior to ligation with the 1.2 kb EcoRI blunt digested Km. The resulting recombinant plasmid was designated pSMSRK.

To delete *embRS* sequence from genomic DNA of *R. gelatinosus*, plasmid pSMSR containing *embR* and *embS* sequence was subjected to digestion with AfeI restriction enzyme to delete a 1.1-kb fragment containing the 3′end of *embR* and the 5′end of *embS* gene. The resulting plasmid was ligated with SmaI-digested Ω cassette to give pSMΔΩ.

A 2-kb PCR DNA fragment containing the *stiK* gene was obtained with primers 156–157 and cloned in pDrive. The fragment was subcloned in KS+ and a Trimethoprim (Tp) cassette was inserted in the EcoRV unique site within *stiK* to inactivate the gene.

The genes reported in this study were cloned in the PCR cloning vector pDrive and inactivated as described in Table S1. Primers are shown in Table S2.

### Deciphering biofilms architecture

A volume of 250 μL of the final overnight subculture adjusted to an OD_600 nm_ of 0.01 (approximately 10^6^ CFU/mL) was added to the wells of a polystyrene 96-well microtiter plate (Greiner Bio-one, France) with a μclear® base (Polystyrene, thickness of 190 ± 5 μm) which allowed for high resolution imaging as described previously (Bridier et al. 2011). After 1 h of adhesion at 30°C, the wells were rinsed with malate growth medium in order to eliminate any nonadherent bacteria before being refilled with 250 μL fresh medium. The plate was then incubated for 12–72 h at 30°C before examination under a confocal microscope. Biofilms were observed using a Leica SP2 AOBS inverted confocal laser scanning microscope (CLSM, Leica Microsystems, France) at the MIMA2 microscopy platform (http://voxel.jouy.inra.fr/mima2) as described previously (Bridier et al. 2011). Bacterial cells were tagged fluorescently in green with Syto9 (1:1000 dilution in the growth medium from a Syto9 stock solution at 5 mmol/L in DMSO; Invitrogen, France), a nucleic acid marker. In some wells, in addition to the green bacterial labeling, polysaccharides within the exopolymeric matrix of the biofilm were stained with 50 μL of Alexa Fluor 633 conjugated to Concanavalin A (ConA) (Molecular Probes, Invitrogen Corporation, Carlsbad, CA, USA). After 20 min of incubation in the dark to enable fluorescent labeling of the bacteria, the plate was then mounted on the motorized stage of the confocal microscope. The microtiter plates were scanned using a high resolution ×63/1.4 N.A. oil immersion objective lens. Syto 9 excitation was performed at 488 nm with an argon laser, and the emitted fluorescence was recorded within the range 500–600 nm in order to collect Syto9. To generate 3D images of the biofilms, Z-image series with a 1 mm step were acquired in three different wells for each condition. Easy 3D blend projections of immersed biofilms were reconstructed from Z-series images using IMARIS v7.0 software (Bitplane, Switzerland).

### RNA extraction and RT-PCR

Total RNA from WT and ΔEmbRS mutant cells grown photosynthetically was extracted according to (Steunou et al. 2004). The RNA concentration was determined by absorption at 260 nm. Specific primers were designed to amplify fragments of *R. gelatinosus wcbM, cpsG, gltA, bmfR, embR*, and *pucB* genes. The primers used for RT-PCR are in Table S2. Reverse primers for each gene were annealed to 2 μg of total RNA extracted and extended for 50 min in the presence of 1 mmol/L dNTPs at 55°C using 200 U of reverse transcriptase (RT) (Superscript III, Invitrogen). A volume of 2 μL of the RT reaction was used for the subsequent PCR; each PCR reaction contained 50 U of Taq DNA polymerase (Qiagen Inc., Valencia, CA) and 5% dimethyl sulfoxide. The PCR program included one cycle of 95°C for 1 min, 30 cycles of 94°C for 10 sec, 54°C for 30 sec, 72°C for 30 sec, and a final incubation at 72°C for 10 min. Amplified products were analyzed by electrophoresis in a 1.2% agarose gel.

## Results

### Sequence analysis of *R. gelatinosus embRS* operon

DNA sequence analysis of the regions flanking the PS cluster of *R. gelatinosus* (Liotenberg et al. 2008) revealed the presence of a two-component system encoding genes adjacent to *bchG*-*bchP* operon (Fig. 1A). The first gene *embR* encodes a putative response regulator belonging to the LuxR family. The gene product EmbR (245 aa) displays a DNA-binding, HTH, domain of about 58 amino acids and a REC receiver domain located in the N-terminus of the protein. Downstream *embR*, a 2238 nucleotide gene designated *embS* encodes a putative transmembrane sensor histidine kinase (745 aa) with ten predicted transmembrane helices. BLAST searches revealed similarity between the amino acid sequence of EmbR and diverse members of LuxR family DNA-binding response regulators from various proteobacteria. Interestingly, however, EmbS only shares significant similarity (52% identity and 67% similarity) with a signal transduction histidine kinase protein (Mpe_A1248) from the closest nonphotosynthetic β-proteobacterium *Methylibium petroleiphilum* PM1. Based on sequence homology, we consider that *embS* is primarily limited to the *Rubrivivax* and *Methylibium* species as it has no other significant detectable sequence similarity in the genomes of other bacteria and is then referred to as orphan gene.

### Phenotype analyses of *embRS* mutants

To gain insight into the role of *embRS* two-component system in *R. gelatinosus*, the *embR* and *embS* genes were individually inactivated in the WT strain. In the EmbRK mutant, the EcoNI fragment within the *embR* gene (Fig. 1A) was deleted and replaced by the Km cassette. To inactivate *embS* gene, a Ω cassette was inserted at the unique XmaI site resulting in the EmbSΩ strain. A ΔEmbRS deletion strain, where a 1.1-kb AfeI fragment containing the 3′end of *embR* and the 5′end of *embS* gene was deleted and replaced by the Ω cassette was also generated.

All mutants EmbRK, EmbSΩ, and ΔEmbRS were selected under aerobic conditions. The three mutants were photosynthetic and produced comparable amounts of photopigments to the WT (data not shown), indicating that this system is not involved in photosynthetic gene regulation even though the operon is physically linked to the PGC cluster. Compared to the WT strain, which had smooth and mucoid colony morphology on plates, the ΔEmbRS mutant like EmbRK and EmbSΩ mutants, presented dry, rugose, and rough colony morphology (Fig. 1A). This indicated that the inactivation of either the putative response regulator (*embR*) or sensor kinase (*embS*) was sufficient to induce changes in colony morphology. The double ΔEmbRS null mutant *(*Δ*embRS::* Ω*)* was used for the subsequent experiments described in this article to avoid any cross talk phenomenon known to occur sometimes between partners of different two-component systems. When the ΔEmbRS mutant was grown in liquid under PS conditions, the mutation led to an uncommon spectacular autoaggregation of the cells in a compact tubular mass (Fig. 1B). The ΔEmbRS null mutant showed an autoaggregative and sinking phenotype in liquid culture as opposed to the homogeneous and planktonic growth exhibited by the WT. This phenotype was also observed under respiratory and dark conditions. The mutant was also characterized by increased adherence and attachment to different solid substrates. As shown in Figure 1C, the ΔEmbRS mutant firmly sticks to the toothpick, suggesting a possible increased ability of the mutant to form a biofilm. The percentage of autoaggregation was estimated by measuring the optical density of the cells that remained in suspension and the optical density of the whole culture after disruption of the aggregate with vigorous vortexing. Almost 90% of the ΔEmbRS cells were found in the aggregate and only 10% in suspension (Fig. S1). By contrast, for the WT, less than 1% of cells autoaggregated in the bottom of the culture tube. Complementation of the ΔEmbRS (Fig. 1B) with the *embRS* operon restores the WT phenotype indicating that the observed phenotypes were directly linked to the loss of these genes. Taken together, these data demonstrate that the *embRS* system is active in the WT strain and is somehow involved in colony morphology, autoaggregation, and probably in biofilm formation.

### Effect of mutation in *embRS* on extracellular matrix biosynthesis and biofilm formation

Given the ability of the *embRS* mutants to grow in multicellular community and to autoaggregate, we questioned whether this phenotype can be caused by the production of an extracellular polymeric substance. To test this hypothesis, the ΔEmbRS mutant was grown under PS conditions. Upon formation of cell aggregates, the cultures were heated or exposed to high concentration of alcohol to eliminate the cells. In both cases, a whitish mass corresponding to the extracellular matrix (EPS) that encased the cells was present in the medium (Fig. 1C). The nature of this exopolysaccharide polymer (called Rubgelatan) is currently under investigation.

**Figure 2:**
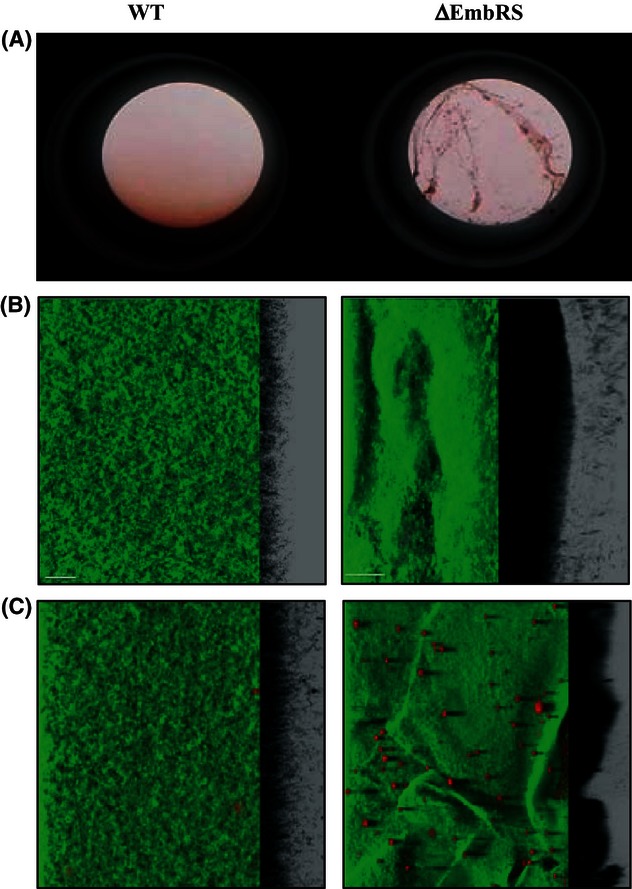
Biofilm formation by ΔEmbRS cells. (A) Digital camera images show macroscopic biofilm structures only in the ΔEmbRS mutant in microplates. (B) Microscopic confocal laser scanning microscopy (CLSM) visualization of the microtiter wells. 12-h biofilms were fluorescently labeled in green with syto 9. (C) Fluorescent (lectin ConA, red) labeling demonstrates the presence of higher amount of exopolysaccharide pockets in the 12-h biofilm of ΔEmbRS mutant compared to the WT.

To determine if the EmbRS two-component system was involved in biofilm formation, we characterized and compared the ability of *R. gelatinosus* WT and ΔEmbRS mutant to adhere and form multicellular layers on polystyrene surfaces. The structure of 24-h biofilm formed by these strains on the surface of wells in a microtiter plate was analyzed (Fig. 2A). Furthermore, microscopic visualization of the microtiter wells by confocal laser scanning microscopy shows an increased biofilm formation of the ΔEmbRS mutant in comparison to the WT (12-h biofilms were fluorescently labeled in green with the nucleic acid marker syto 9) (Fig. 2B). A second labeling with Concanavalin A (fluorescent exopolysaccharide marker) indicates the presence of larger amount of exopolysaccharide pockets scattered within the bacterial structure of the ΔEmbRS mutant compared to the WT (Fig. 2C). These analyses demonstrated that the WT strain was in fact able to form a biofilm on polystyrene microplates. However, the ability of the ΔEmbRS mutant to form a biofilm was significantly increased in the same conditions. Crystal violet staining of ΔEmbRS cells forms aggregate and planktonic suspension confirms the presence of a polymeric material surrounding cells in aggregates (Fig. S2). Interestingly, this material serves as a physical barrier for the aggregated cells, preventing the entry of the free cells in the aggregate microenvironment as is evidenced in the 2 min movie M1 and in Figure S2.

**Figure 3:**
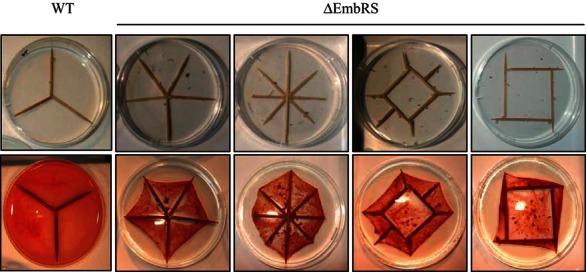
Biofilm formation by ΔEmbRS cells. Formation of bacterial veils around the toothpick scaffolds. Cells were inoculated in the liquid medium and grown photosynthetically in plates. The extracellular matrix polymerization and its anchorage to the toothpicks led to the formation of the polygonal networks. (See movie M2).

The ability of ΔEmbRS mutant to stick to a toothpick in the tube (Fig. 1C) led us to look for a possible role of the toothpicks in guiding polymerization of the extracellular matrix in a planar surface. To this end, ΔEmbRS cell aggregates were scattered within the medium in the plates containing toothpick scaffolds and allowed to grow by photosynthesis overnight. As shown in Figure 3, growth resulted in the formation of conspicuous bacterial veils around the toothpick scaffolds. Cells and the matrix were associated with the toothpicks in large well-arranged polymeric structures. The extracellular matrix polymerization and its anchorage to the toothpicks led to the formation of very organized polygonal networks where cells are gathered. This prompted us to question about the rearrangement of the biofilm and to continuously follow its formation to capture the details of this rearrangement. As shown in movie M2 and in Figure S3, the cells/matrix grew until the matrix was associated with the plate borders; then rearrangement of the biofilm started when the matrix spontaneously relaxed. The shrinking of the biofilm continued until the veil was in contact with the top of the toothpicks, generating the organized polygonal network. These data confirm that the extracellular matrix produced by the ΔEmbRS mutant allows cell assembly, adhesion, and gathering. To our knowledge, such ability to form polygonal shapes has never been previously reported for bacterial biofilms.

As the ΔEmbRS mutant produces dramatically more extracellular matrix than WT strain, we conclude that the EmbRS system is involved in regulating the production of the extracellular matrix and in biofilm formation. We hypothesize that EmbRS acts as a negative regulator that either directly or indirectly represses the expression of genes involved in the biosynthesis of the extracellular substance.

### Large-scale transposon mutagenesis reveals a new genetic locus (psc1) important for biofilm formation

**Figure 4:**
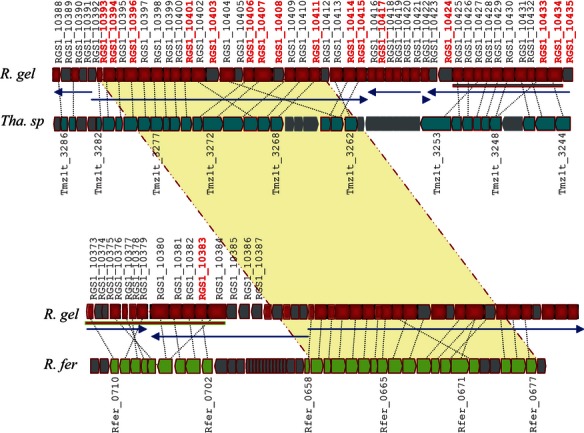
Polysaccharide synthesis gene cluster *psc1*. There is 75-kb chromosomal region synteny between *Rubrivivax gelatinosus*, *Thauera sp. MZ1T*, and *Rhodoferax ferrireducens T118*. The genes identified by transposition in this study are shown in red. The conserved genes in the three species are shown in yellow. Arrows correspond to putative operons.

Based on the Δ*EmbRS* null mutant phenotypes described above, we predicted that the inactivation of genes functioning together with EmbRS in controlling biofilm formation would lead to strains with either reduced or abolished ability to autoaggregate and to form a biofilm. Similarly, the disruption of genes required for the biosynthesis of the extracellular polymer in the rugose genetic Δ*EmbRS* background should yield smooth colonies and mutants that regain, partially or totally, the planktonic lifestyle. A Tn5-transposon insertion library was created using the ΔEmbRS mutant (5000 mutants). The difference in colony morphology provided an easy screening method for the selection of phenotypic reversion mutants. Many colonies exhibiting smooth or quasi-smooth and mucoid morphologies were identified. As shown in Figure S4, selected mutants resulting from this screen exhibited the expected phenotypes (reduced or abolished ability to autoaggregate on plates S4A and in liquid S4B) compared to the ΔEmbRS mutant parental strain. Mapping of the Tn5 insertions in the genome of 40 mutants was undertaken. The genomic regions flanking the transposon insertion sites were determined by sequencing and the genes and loci were designated based on the Genoscope *R. gelatinosus* S1 RGS1 draft genome sequence. This analysis revealed that most clones corresponded to distinct insertion events. As a result, 32 different genes were identified. Twenty-five insertions were found in genes located within a 75 kb chromosome region designated *psc1* for polysaccharide synthesis cluster 1 (Fig. 4). This cluster encompasses 63 genes; 42 genes encode proteins involved in polysaccharide (capsular, lipo- and exopolysaccharide) biosynthesis and export and 21 open reading frames (ORFs) encode proteins of unknown function or with no significant protein database match. Table 1 provides the sequence data information about the cluster and the inactivated genes identified in the course of this study. Among the 25 insertions, two genes code for the capsular biosynthesis proteins CapK and CapL, five genes code for different glycosyltransferases, and three genes encode proteins involved in polysaccharide export (*wza, wzy*, and *wzx*). Genes involved in the lipopolysaccharides biosynthesis pathway were also identified in the screen in addition to four ORFs encoding proteins of unknown function. Synteny search and blast sequence analyses of the 75-kb DNA fragment unexpectedly revealed an extraordinary conservation between *R. gelatinosus* and *Thauera sp. MZ1T*. Gene content and organization were well conserved in both species (Fig. 4 and Table 1). *Thauera sp*. is known as a floc-forming β-proteobacterium that constitutively produces large amounts of extracellular polysaccharides (Allen et al. 2004). Nevertheless, in *Thauera*, the genetic determinants responsible for such production remain still unknown. A less pronounced conservation of these genes and of their organization was also observed with *Rhodoferax ferrireducens T118* and *Azoarcus sp. BH72*.

**Table 1:** Genes in the *psc1* cluster and their homologues from *Thauera sp*. and *Rhodoferax ferrireducens*

*Rubrivivax gelatinosus*	*Thauera sp. MZ1T*	*R. ferrireducens T118*	Predicted function
RGS1_10373	Tmz1t_1602	Rfer_0710	Peptidase S1 and S6 chymotrypsin (*mucD*/*algY*)
RGS1_10374	*Tmz1t_3283*	–	Conserved protein of unknown function
RGS1_10375	*–*	–	Protein of unknown function
RGS1_10376	*–*	Rfer_0706	Protein of unknown function
RGS1_10377	–	Rfer_0707	Protein of unknown function
RGS1_10378	*Tmz1t_1428*	Rfer_0708	Hpr(Ser) kinase/phosphatase
RGS1_10379	*Tmz1t_1427*	Rfer_0709	Conserved protein of unknown function
RGS1_10380	*Tmz1t_1628*	Rfer_0705	Tetratricopeptide TPR_2 PEP-CTERM TPR-repeat lipoprotein (*algB*)
RGS1_10381	Tmz1t_1599	Rfer_0704	Two component, transcriptional regulator PEP-CTERM-box (*wcaJ*)
RGS1_10382	Tmz1t_1598	Rfer_0703	Two component, sensor protein, PEP-CTERM histidine kinase (*KinB*)
*RGS1_10383	Tmz1t_1597	Rfer_0702	Undecaprenyl-phosphate galactosephosphotransferase
RGS1_10384	*Tmz1t_1628*	Rfer_0705	Tetratricopeptide TPR_2 PEP-CTERM TPR-repeat lipoprotein
RGS1_10385	–	–	Protein of unknown function (PEP_exosortase)
RGS1_10386	Tmz1t_3271	*Rfer_0670*	Putative phosphatidylinositol alpha-mannosyltransferase
RGS1_10387	–	*–*	Conserved exported protein of unknown function
RGS1_10388	Tmz1t_3286	*–*	Conserved exported protein of unknown function
RGS1_10389	Tmz1t_1621	*Rfer_3187*	Putative ABC-type transport system permease component
RGS1_10390	Tmz1t_3285	Rfer_3186	ABC transporter, ATP-binding protein
RGS1_10391	Tmz1t_1624	–	UBA/THIF-type NAD/FAD-binding fold (fragment)
RGS1_10392	Tmz1t_1623	–	UBA/THIF-type NAD/FAD-binding protein
*RGS1_10393	Tmz1t_3282	Rfer_0658	Polysaccharide export protein (2) (*wza*)
*RGS1_10394	Tmz1t_3281	Rfer_0659	Lipopolysaccharide biosynthesis
RGS1_10395	Tmz1t_3280	Rfer_0660	Nonspecific protein-tyrosine kinase (*epsB*)
*RGS1_10396	Tmz1t_3279	Rfer_0661	Exported protein of unknown function
RGS1_10397	Tmz1t_3278	Rfer_0662	ATPase
RGS1_10398	Tmz1t_3277	Rfer_0663	UDP-*N*-acetylglucosamine 2-epimerase (*epsC*)
RGS1_10399	Tmz1t_3276	Rfer_0664	Polysaccharide deactylase family protein, PEP-CTERM (*nodB*)
RGS1_10400	Tmz1t_3275	Rfer_0665	FemAB-related protein, PEP-CTERM system-associated (*femA*)
*RGS1_10401	Tmz1t_3274	Rfer_0672	Glycosyltransferase, group 1
RGS1_10402	Tmz1t_3270	Rfer_0666	Conserved membrane protein of unknown function
*RGS1_10403	Tmz1t_1116	Rfer_0667	Protein (*capL*)
RGS1_10404	Tmz1t_3262	Rfer_2500	Membrane bound *O*-acyl transferase MBOAT family protein (*algI*)
RGS1_10405	–	–	Protein of unknown function
*RGS1_10406	Tmz1t_3273	Rfer_0668	Glycosyltransferase, group 1 (2)
*RGS1_10407	Tmz1t_3272	Rfer_0669	Asparagine synthase, glutamine-hydrolyzing (*wbpS*)
*RGS1_10408	Tmz1t_3271	Rfer_0670	Glycosyltransferase, group 1
RGS1_10409	Tmz1t_3271	Rfer_0675	Glycosyltransferase, group 1
*RGS1_10410	Tmz1t_3269	Rfer_0676	Capsular polysaccharide biosynthesis protein CapK
*RGS1_10411	Tmz1t_3268	Rfer_0677	Conserved membrane protein of unknown function (*wzy*)
RGS1_10412	–	–	Exported protein of unknown function
RGS1_10413	Tmz1t_3262	Rfer_2500	Putative poly(beta-d-mannuronate) *O*-acetylase transferase MBOAT (*algI*)
*RGS1_10414	Tmz1t_3264	–	Conserved exported protein of unknown function
*RGS1_10415	Tmz1t_3263	Rfer_0670	Glycosyltransferase group 1 (2)
RGS1_10416	Tmz1t_0872	*Rfer_3694*	d,d-heptose 1,7-bisphosphate phosphatise (*gmhB*)
*RGS1_10417	Tmz1t_2875	Rfer_0712	d-glycero-d-manno-heptose 1-phosphate guanosyltransferase (*wcbM*)
RGS1_10418	Tmz1t_3441	*Rfer_3874*	Phosphoheptose isomerase (*gmhA*)
RGS1_10419	–	–	d-glycero-d-manno-heptose 7-phosphate kinase (*wcbL*)
RGS1_10420	Tmz1t_3804	Rfer_0668	Protein of unknown function (*mshA*)
RGS1_10421	–	–	Protein of unknown function
RGS1_10422	–	Rfer_0685	Membrane protein of unknown function (*oatA*)
RGS1_10423	Tmz1t_3247	Rfer_0669	Putative asparagine synthase (*wbpS*)
*RGS1_10424	–	–	Exported protein of unknown function
RGS1_10425	Tmz1t_3252	Rfer_0687	Glycosyltransferase family 2
RGS1_10426	Tmz1t_3251	–	Conserved protein of unknown function
RGS1_10427	Tmz1t_3253	–	Sulfatase (*wbbL*)
RGS1_10428	Tmz1t_3250	Rfer_0771	Glycosyltransferase family 2
RGS1_10429	Tmz1t_3249	Rfer_0678	Polysaccharide deacetylase (*pgaA*)
RGS1_10430	Tmz1t_3248	Rfer_0687	Glycosyltransferase family 2
RGS1_10431	Tmz1t_3246	–	Lipopolysaccharide biosynthesis protein-like (*rgpF*)
RGS1_10432	Tmz1t_3245	–	Conserved protein of unknown function
*RGS1_10433	Tmz1t_3248	Rfer_0687	Glycosyltransferase (2)
*RGS1_10434	Tmz1t_3244	Rfer_0691	Putative polysaccharide biosynthesis protein (*wzxC*)
*RGS1_10435	Tmz1t_3265	Rfer_0683	Asparagine synthase (Glutamine hydrolyzing) (*wbpS*)
RGS1_10436	–	–	Conserved exported protein of unknown function

* Genes with transposon insertion.

### Transposon mutagenesis revealed a second locus (psc2) associated with a gluey and sticky phenotype in the ΔEmbRS background

**Figure 5:**
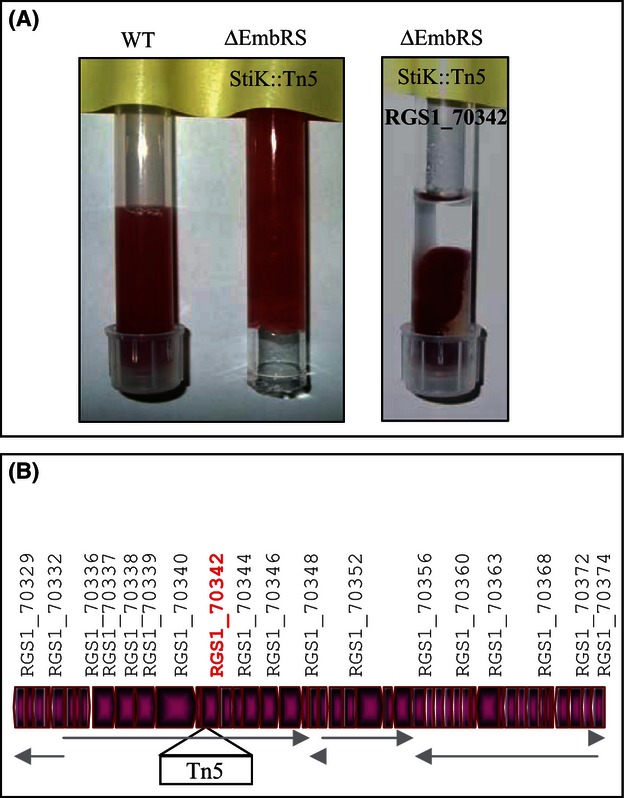
Phenotype of the ΔEmbRS-Stik mutant. (A) Cells (WT, ΔEmbRS-STiK::Tn5, and the complemented strain ΔEmbRS-STiK::Tn5 + RGS1_70342) were grown photosynthetically and tubes were turned upside down to illustrate the StiK phenotype. (B) The polysaccharide synthesis gene cluster *psc2*. The *stik* gene (RGS1_70342), responsible for this phenotype, identified by transposition in this study is shown in red within the 50 kb polysaccharide biosynthesis genes cluster 2. Arrows correspond to putative operons.

In the series of transposon mutants that recovered a partial planktonic growth phenotype, the mutant ΔEmbRS-RGS1_70342 presented a heterogeneous planktonic growth phenotype when grown in liquid as opposed to the autoaggregative phenotype of the ΔEmbRS strain. Surprisingly, however, the double mutant exhibited a sticky phenotype and firmly stuck to the tube surface. As shown in Figure 5, even if the tube was turned upside down, the culture hung in the tube and stuck firmly inside the tube. The nature and structure of the synthesized extracellular matrix in this strain is under investigation. The RGS1_70342 (*stiK*) gene encodes a putative protein of unknown function with no significant database matches and no homology to any previously reported sequence. The exact function of this gene and its effect on polysaccharide biosynthesis remain to be established. The RGS1_70342 gene is found within a 50 kb region (designated *psc2*) in which many (37) genes encode proteins and enzymes predicted to be involved in polysaccharide (capsular, lipo- and exopolysaccharide) biosynthesis (Fig. 5 and Table 2). Fifteen ORFs within the cluster have unknown functions. Sequence analyses suggest, however, that they encode proteins, very likely involved in carbohydrate modifications, given that most of them exhibit domains with homologies to known carbohydrate-sulfo-transferases or glycosyltransferases. Genes involved in capsular polysaccharide transport (*kps* genes) were also identified in the cluster (Table 2). To verify whether the phenotype observed in the mutant resulted from a loss of function of *stiK* gene, we inactivated this gene in the ΔEmbRS background by homologous recombination. The resulting strain was similar in phenotype to the selected transposon mutant. Furthermore, complementation of the ΔEmbRS-StiK strain with *stiK* gene restores the autoaggregative ΔEmbRS phenotype (Fig. 5).

**Table 2:** Genes in the *psc2* cluster and their homologues from *Thauera sp*. and *Rhodoferax ferrireducens*

*Rubrivivax gelatinosus*	*Thauera sp. MZ1T*	*R. ferrireducens T118*	Predicted function
RGS1_70329	–	Rfer_1904	PfkB domain protein (modular protein)
RGS1_70330	–	–	Glycerol-3-phosphate cytidylyltransferase
RGS1_70331	–	–	Protein of unknown function
RGS1_70332	Tmz1t_1646	Rfer_3028	ATP-dependent RNA helicase rhlE
RGS1_70333	Tmz1t_3794	–	Putative glycosyltransferase, family 4; putative Phospho-*N*-acetylmuramoyl-pentapeptide-transferase
RGS1_70334	–	–	Putative polysialic acid transport protein kpsM
RGS1_70335	Tmz1t_1019	Rfer_3558	Polysialic acid transport ATP-binding protein KpsT
RGS1_70336	–	–	Capsular polysaccharide ABC transporter KpsE
RGS1_70337	Tmz1t_3790	Rfer_2676	Capsular polysaccharide synthesis KpsD protein
RGS1_70338	–	–	Protein of unknown function
RGS1_70339	–	–	Protein of unknown function
RGS1_70340	Tmz1t_3252	–	Protein of unknown function
RGS1_70341	–	–	Protein of unknown function
*RGS1_70342	–	–	Protein of unknown function (StiK)
RGS1_70343	–	–	Protein of unknown function
RGS1_70344	Tmz1t_3252	Rfer_1256	Protein of unknown function
RGS1_70345		–	Exported protein of unknown function
RGS1_70346		–	Protein of unknown function
RGS1_70347	Tmz1t_2139	Rfer_1237	Putative GDP-mannose 4,6-dehydratase
RGS1_70348		–	Putative methyltransferase FkbM family
RGS1_70349		–	Protein of unknown function
RGS1_70350	Tmz1t_3310	Rfer_1235	GDP-l-fucose synthase
RGS1_70351		–	Protein of unknown function
RGS1_70352		Rfer_1256	Putative glycosyltransferase
RGS1_70353	Tmz1t_3248	–	Protein of unknown function
RGS1_70354		–	Exported protein of unknown function
RGS1_70355		–	Protein of unknown function
RGS1_70356	Tmz1t_3899	–	Putative glycosyltransferase group 1
RGS1_70357	Tmz1t_3958	Rfer_0670	Putative glycosyltransferase
RGS1_70358	Tmz1t_3263	–	Glycosyltransferase, group 1 family protein
RGS1_70359	Tmz1t_3811	Rfer_2678	dTDP-4,deoxyrhamnose 3,5 epimerase
RGS1_70360	Tmz1t_3810	Rfer_2677	dTDP-glucose pyrophosphorylase
RGS1_70361	Tmz1t_2138	Rfer_0714	TDP-rhamnose synthetase, NAD(P)-binding
RGS1_70362	Tmz1t_2139	Rfer_0715	dTDP-glucose 4,6 dehydratase, NAD(P)-binding
RGS1_70363	Tmz1t_1131	Rfer_2679	Polysaccharide biosynthesis protein (capD)
RGS1_70364	Tmz1t_1126	Rfer_0702	Putative sugar transferase
RGS1_70365	Tmz1t_3256	Rfer_1253	Spore coat polysaccharide biosynthesis protein spsC
RGS1_70366	–	–	Methyltransferase type 11
RGS1_70367	Tmz1t_1125	Rfer_0670	Glycosyltransferase group 1
RGS1_70368	Tmz1t_1801	Rfer_0668	Glycosyltransferase group 1
RGS1_70369	Tmz1t_2208	Rfer_0690	Conserved protein of unknown function
RGS1_70370	–	–	Putative export of O-antigen and teichoic acid
RGS1_70371	Tmz1t_1130	Rfer_1239	DegT/DnrJ/EryC1/StrS aminotransferase
RGS1_70372	Tmz1t_3784	Rfer_0690	Putative acetyltransferase
RGS1_70373	–	Rfer_1244	Oxidoreductase domain protein

* *stiK* gene isolated upon transposon mutagenesis. The genes involved in fucose and rhamnose synthesis are highlighted.

### Mutations in the GDP-d-mannose biosynthesis pathway partially restore the smooth phenotype and provide insight into the nature of the extracellular matrix

In two ΔEmbRS-Tn5 mutants that partially recovered a smooth and planktonic phenotype, the identified genes *cpsG* (*algC*) and *wcbM* (*algA*) encode a putative phosphomannomutase and a d-glycero-d-manno-heptose 1-phosphate guanosyltransferase, which catalyze the conversion of α-d-mannose 1-phosphate to d-mannose 6-phosphate and to Guanosine diphosphate (GDP)-mannose, respectively. These enzymes are required for the synthesis of GDP-mannose, the precursor of many polysaccharides in bacteria. The GDP-mannose is then converted to GDP-l-fucose in the de novo biosynthesis pathway, via three enzymatic reactions carried out by a GDP-d-mannose dehydratase and a bifunctional GDP-l-fucose synthase. Genes encoding both enzymes are found in the genome of *Rubrivivax* in the 50 kb cluster (*psc2*) (RGS1_70347 and RGS1_70350) and suggest the presence of fucose or its derivatives in the polysaccharides of this bacterium. Taken together these genetic and genomic data clearly suggest that these loci (*psc1* and *psc2*) are involved in the biosynthesis and export of the extracellular biopolymer, most likely polysaccharides, and are thereby required for flocculation and biofilm formation in *R. gelatinous* and in *Thauera sp. MZ1T*.

### BmfS a 7TMR-HD receptor and BmfR response regulator, potential additional two-component regulatory genes controlling biofilm formation

The ΔEmbRS-RGS1_30021 mutant recovered quasi-WT smooth colony morphology and a planktonic liquid growth lifestyle. Mapping of the mutation indicated that the transposon insertion occurred within a two-component sensor signal transduction histidine kinase RGS1_30021 (annotated BmfS for biofilm and matrix formation). Analyses of the sequence revealed that, in addition to the cytoplasmic histidine kinase domain this protein possesses a putative 7TMR signaling domain consisting of 7-transmembrane helices. In bacteria, 7TMRs signaling proteins usually possess extracellular globular domains predicted to bind ligands such as carbohydrates. Domains and secondary structure prediction search suggest that BmfS has a large periplasmic domain in its N-terminus. A carbohydrate-binding domain with a putative *O*-glycosyl hydrolase activity is embedded within this periplasmic predicted large domain (Fig. S5A). A gene *bmfR* (RGS1_30022) localized downstream of *bmfS*, encodes a response regulator with a DNA-binding (HTH-LuxR) domain and a REC receiver domain. This may indicate that BmfR is the cognate response regulator of the BmfS sensor kinase. Interestingly, the BmfR sequence shares very high homology (43% identity and 80% similarity) with the EmbR sequence. Noteworthy, BmfR sequence shows a 22 amino acid deletion compared to EmbR (Fig. S5B). To confirm the involvement of *bmfR* in the biofilm formation, a ΔEmbRS-ΔBmfR double mutant was constructed. This mutant exhibited the same phenotype as ΔEmbRS-RGS1_30021. Complementation of the ΔEmbRS-ΔBmfR strain with *bmfR* gene restores the autoaggregative ΔEmbRS phenotype (data not shown) confirming the role of BmfSR system in biofilm formation.

Furthermore, it was reported in gram-positive bacteria that the 7TMR-HD gene is always specifically located adjacent to the *phoH* and *ybeY* genes (Anantharaman and Aravind 2003). Interestingly, synteny search demonstrates that in *R. gelatinosus* and the closest species *M. petroleiphilum* PM1 and *L. cholodnii*, *bmfSR* operon is located immediately upstream the *phoH* and *ybeY* cluster as reported in gram-positive bacteria. Altogether, these data suggest that BmfS is a 7TMR-HD membrane-associated receptor histidine kinase, positively involved in the biosynthesis of the extracellular matrix in the ΔEmbRS mutant. Given that its inactivation abolishes synthesis of the extracellular matrix in the ΔEmbRS, this suggests that the BmfSR system should be downstream of EmbRS in the signal transduction pathway and should be repressed by EmbRS in the WT background. Blast analyses of BmfSR revealed that homologues of this system might be also present in many other bacteria including *Bordetella* (BN115_2398)*, Cupriavidus* (Rmet_4987)*, Ralstonia* (H16_B2298), *Methylibium* (Mpe_A3238), and *Leptothrix* (Lcho_3951). No homologue of BmfSR was found, however, in the *Thauera* genome.

### Genetic screening suggests the involvement of second messenger signaling c-di-GMP in biofilm induction

The bacterial intracellular signaling molecule cyclic di-GMP (c-di-GMP) was identified as a factor controlling the biofilm formation in pathogenic and nonpathogenic bacteria (Cotter and Stibitz 2007; Tamayo et al. 2007; Hengge 2009). Mutation in di-GMP cyclase would be expected to reduce the intracellular levels of c-di-GMP and potentially lead to a decreased capacity for biofilm formation.

In a second ΔEmbRS-Tn5 mutant that recovered quasi-WT smooth colony morphology and a planktonic growth phenotype, the transposon mapped within the RGS1_40251 gene (*bdcA*), which encodes a putative protein with a GGDEF domain. Complementation of the ΔEmbRS-RGS1_40251 strain with pBbdcA plasmid bearing the *bdcA* gene restores the autoaggregative phenotype of ΔEmbRS indicating *bdcA* is somehow involved in ΔEmbRS phenotype. The protein sequence analysis showed that this gene encodes for a diguanylate cyclase carrying a HAMP domain and the conserved GGEEF residues in its active site. BdcA exhibits similarity with diguanylate cyclases from many bacteria. The highest homology (46% identity and 60% similarity) was recorded with a diguanylate cyclase (Pfl01_3550) from *Pseudomonas fluorescens* Pf0-1. The diguanylate cyclase activity of BdcA and the c-di-GMP level remain to be determined, however, the phenotype of ΔEmbRS-RGS1_40251 mutant is consistent with a decrease in the c-di-GMP levels, which will promote a planktonic over a sessile lifestyle and a reduction of biofilm formation in this strain. It is tempting therefore to suggest a putative model where c-di-GMP signaling controls the extracellular matrix (polysaccharides) production and thereby biofilm formation in *R. gelatinosus*.

### Other genes that affect the colony morphology and cells autoaggregation behavior

Using this suppressor strategy, we also identified mutations in other genes that modify the autoaggregative phenotype of the ΔEmbRS null mutant. Among these genes, we found that mutation in RGS1_10407 led also to a partial planktonic mode of growth and different colony morphology of the mutant compared to ΔEmbRS null mutant. RGS1_10407 encodes a putative protein with conserved domains of the glutamine-dependent amidotransferase family. The amino acid sequence shares high homology (37% identity and 55% similarity) with the glutamine amidotransferase protein WbpS from *P. aeruginosa*. In *P. aeruginosa*, WbpS catalyzes the biosynthesis of uronamides by transfer of ammonia to the carboxylate moiety of the corresponding uronic acid and it is therefore required for lipopolysaccharide O-antigen biosynthesis (King et al. 2010). It is well known that lipopolysaccharides are involved in colony morphology (rugose vs. smooth) in many bacteria. The disruption of RGS1_10407 in ΔEmbRS null mutant background may thus modify the colony morphology of the mutant by affecting the synthesis and/or the structure of the lipopolysaccharides in this strain.

Another mutation that partially suppressed the autoaggregative phenotype and restored a partial planktonic lifestyle of the ΔEmbRS mutant mapped within the RGS1_10841 gene (FO082880). This gene encodes for PilM, a protein of the type IV pili biogenesis machinery (Ayers et al. 2009). Type IV pili are involved in many processes in bacteria, including twitching motility, adherence, and biofilm formation (Mandlik et al. 2008; Mikkelsen et al. 2011). It is therefore likely that the inactivation of PilM in ΔEmbRS null mutant impact indirectly on the aggregative phenotype by affecting the pili formation and not the extracellular matrix synthesis.

### Expression of genes involved in the matrix synthesis in the ΔEmbRS deficient mutant

**Figure 6:**
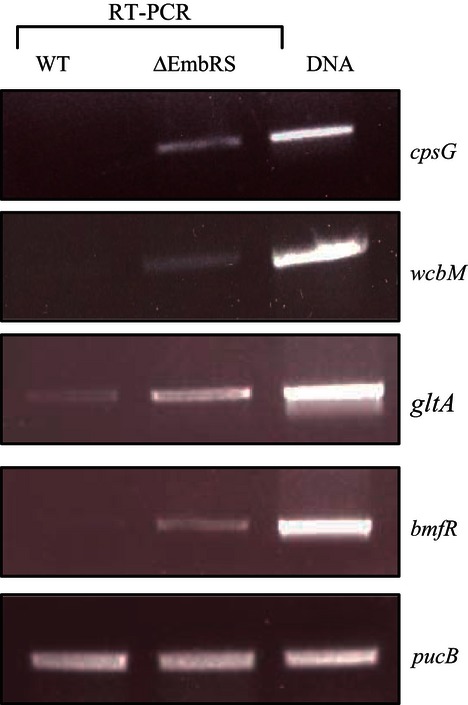
Expression profiles of genes regulated by EmbRS. RT-PCR experiment showed an increase in the transcript level of all regulated genes in the ΔEmbRS mutant compared to the wild type (WT). *pucB* encoding the light harvesting II β-subunit was used as a control.

To validate the role of the EmbRS system as a negative regulator of genes involved in the extracellular matrix synthesis and biofilm formation, RT-PCR analysis was performed on RNAs obtained from the WT and the ΔEmbRS null mutant. Among the genes identified in the genetic screen that might be negatively regulated by EmbRS, we focused our study on the expression profile of three genes *cpsG* (RGS1_80151), *wcbM* (RGS1_10417), *gltA* (RGS1_10433) involved in polysaccharide and probably the extracellular matrix biosynthesis and on the two-component system regulator encoding gene *bmfR* (RGS1_30022) that seems to positively control the biofilm formation. As shown in Figure 6, all four genes show very low level of expression in the WT background. By contrast, these genes exhibit a significantly increased expression level in the ΔEmbRS strain indicating that they were negatively regulated by the EmbRS two-component system. These data suggest that EmbRS should repress polysaccharide biosynthesis and corroborate the genetics data indicating that this system represses biofilm formation in the WT background.

### From the bottom to the surface, *embRS* acquisition might indicate an adaptation of R. gelatinosus to a photosynthetic lifestyle

**Figure 7:**
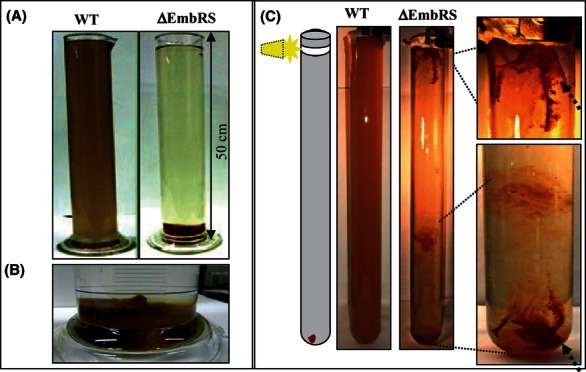
*embRS* deletion results in fast sinking of bacteria. (A) PS grown ΔEmbRS cells display an autoaggregative growth and sink to the bottom of the tube compared to planktonic growth of the WT cells. (B) Transferred aggregated cells to a new tube remain compact and sink. (C) Effect of EmbRS mutation on PS growth. Tubes were wrapped in aluminum foil and lighted only from the top as in the cartoon. The WT cells found throughout the length of the tube. ΔEmbRS cells autoaggregate and sink, their PS growth was limited. The arrows on the magnified pictures show the ΔEmbRS cells, grown at the top of the tube and embedded in the veil that sinks later to the bottom of the tube.

Bacteria have developed diverse tools and mechanisms to move and swim toward favorable niches. Among these, chemotaxis, flagella, and pili allow bacteria to swim in aqueous environments or swarm and twitch on solid surfaces toward nutrients (Jarrell and McBride 2008). Photosynthetic cyanobacteria and other proteobacteria, make use of gas vesicles to control flotation, which allows them to reach the light and/or nutrient-rich euphotic zone in the water column (Jarrell and McBride 2008). By opposition to gas vesicles, polysaccharides overproduction can induce autoaggregation of cells and result in the migration of bacteria from the surface to the depth of the water column. This phenomenon is illustrated in Figure 7A. The ΔEmbRS null mutant cells formed aggregates in liquid cultures and sank to the bottom of the tube. By contrast, in the WT, the EmbRS system prompted a planktonic lifestyle and allowed cells to grow all along the medium column. When transferred from the culture medium to a new tube, the aggregate remained compact and sank rapidly to the bottom of the tube (Fig. 7B). In deep aquatic environment, this phenomenon could be disadvantageous for the mutant, because sinking cells would be deprived of the sunlight needed for photosynthesis. To test the impact of polysaccharides overproduction and autoaggregation on the photosynthetic growth of the ΔEmbRS mutant, we compared the growth ability of the WT and the mutant under low-light conditions in 50-cm height tubes. In these tubes, cells from both strains were inoculated in the medium; the tubes were then wrapped in aluminum foil and lighted only from the top to mimic an aquatic natural environment. These cells (both WT and mutant) cannot grow unless they move from the bottom to get light at the top of the tube. After 3 days, the aluminum wrapping was removed to check the growth of the cells. As shown in Figure 7C, the WT cells were found throughout the length of the tube. They have probably grown by light at the top of the tube (where light is available) and then moved throughout the length of the tube in the medium. By contrast, ΔEmbRS cells trapped in the polysaccharide matrix exhibited lower growth compared to the WT. A maximum of photosynthetic growth was observed in the top of the tube where light intensity was maximal. In this area, a red “veil” composed of photosynthetic cells and the extracellular matrix was attached to the top of the tube. This indicated that cells from the ΔEmbRS mutant were able to move from the bottom toward the light source, adhere and establish a biofilm to set up photosynthesis. The veil ended in the bottom of the tube few hours later. Aggregated, but less pigmented cells were also present in the growth medium as shown in Figure 7B. This experiment demonstrated that ΔEmbRS cells can come off the aggregate and move close to light. Nevertheless, autoaggregation resulted in fast sinking and appears to be prejudicial to the photosynthetic growth of the ΔEmbRS mutant. Thus, in order to overcome the undesirable polysaccharide overexpression, we speculated that *R. gelatinosus* might have evolved the EmbRS two-component system as a way to control the production of these polysaccharides. Planktonic life style can therefore take place through the EmbRS activity and in concert with flagella and pili, thereby allowing *R. gelatinosus* to migrate from the aphotic depth to the euphotic zone and to set up photosynthesis.

## Discussion

Microbial production of polysaccharides and attachment to surfaces occur both in natural and engineered systems and are responsible for various types of biofouling and viscous bulking (van den Akker et al. 2010; Xiong and Liu 2010; Flemming 2011). This report provides evidence supporting the involvement of the two-component system EmbRS in the repression of polysaccharides production and biofilm formation in *R. gelatinosus*. The inactivation of *embRS*: (i) affects the morphology of colonies and originates in dry and rough colonies, (ii) results in autoaggregation of cells and increased adherence and attachment to solid surfaces, (iii) increases extracellular matrix production and biofilm formation. Analyses of transposon mutants that recovered quasi-WT phenotype revealed that most mutations mapped within a polysaccharide biosynthesis and transport encoding cluster. The gene organization of this cluster is fully conserved only in one bacterium, *Thauera sp. MZ1T*. Although this has not yet been directly tested, we predict that this cluster would be also involved in polysaccharides synthesis and floc formation in *Thauera sp*. In support of this, however, are the facts that the locus is well conserved between these species and that *Thauera sp*. is a flocculating bacterium that produces high amounts of extracellular polysaccharides (Allen et al. 2004). It is interesting that, *embRS* operon is absent in the *Thauera sp*. genome; this may account for the constitutive and high production of exopolysaccharides by this particular species. The transposon genetic screen also revealed a di-GMP cyclase and a second two-component system that is involved in biofilm formation in the EmbRS background. The BmfRS system encodes for a putative LuxR-type response regulator and a 7TMR-HD histidine kinase that, in contrast to EmbRS, should activate the expression of genes involved in extracellular matrix production and biofilm formation in *R. gelatinosus*. Although the exact function of these regulators BdcA and BmfRS and their impact on polysaccharide biosynthesis gene expression remain to be established, our findings demonstrate a genetic link between EmbRS, BmfRS, BdcA, and the autoaggregative phenotype of the EmbRS mutant. The data indicate that EmbRS is a master two-component regulator that represses biofilm formation in *R. gelatinosus*, probably in concert with BmfRS and BdcA. A first proposed regulatory model for extracellular matrix production and biofilm formation by EmbRS is shown in Figure S6.

### On the importance of controlling biofilm formation in phototrophs

One may question about the purpose of EmbRS system to repress the biofilm formation despite the benefit and advantage that this structure can confer. As stated above, in natural environment, aggregated photosynthetic microorganisms sinking in the column water down to the aphotic zone, characterized by limited light, will be deprived from sunlight and unable to photosynthesize. Conversely, a planktonic lifestyle is expected to provide a significant advantage over a sessile lifestyle, as photosynthetic microorganisms can move toward the euphotic zone where light will promote cell development. Consequently, it is tempting to speculate that the occurrence of the EmbRS system in *R. gelatinosus* or other biofilm repressors in other phototrophic bacteria may be indicative of a need for decreased quantities of extracellular matrix production and the inhibition of biofilm formation to prevent the fast-sinking aggregates and favor photosynthetic planktonic growth. Nonetheless, making biofilm and aggregates will be advantageous in case of stresses such as limited light or nutrients. Under these conditions, a stress signal triggering the biosynthesis of the extracellular matrix and biofilm formation (Nadell et al. 2008; Ueda and Wood 2009; Yildiz and Visick 2009) would favor sinking to the nutrients rich aphotic zone. It would be a valuable asset for photosynthetic microorganisms to switch between the two zones to get benefits from light supplied in the top, and from nutrients supplied from the depth under limited light conditions. It is noteworthy that *R. gelatinous* WT strain produces an extracellular matrix in the late stationary growth phase. This led to the formation of cell aggregates (less abundant compared to EmbRS mutant) indicative of intercellular adhesion and biofilm formation, probably as a result of nutrient-limited environment (Fig. S7). Acquisition of EmbRS two-component system would allow the control of the switch between the planktonic and aggregative lifestyles.

### *embRS* acquisition: a scenario that may indicate an adaptation to a photosynthetic lifestyle

In photosynthetic proteobacteria, including *Rubrivivax*, most genes required for photosynthesis are localized within a single PGC (Igarashi et al. 2001; Liotenberg et al. 2008). This has facilitated horizontal gene transfer between bacteria, and it is well accepted that *R. gelatinosus* had acquired the PGC by horizontal transfer of the photosynthetic genes from a purple bacterium in the α-subclass (Igarashi et al. 2001). It is possible that location of the *embSR* genes downstream the PGC is simply coincidental. However, it is tempting to speculate on the *embRS* role and acquisition in relation with photosynthetic growth of this bacterium in the water column. If *R. gelatinosus* ancestor was a flocculating bacterium that acquired the PGC cluster subsequently to acquiring the polysaccharide clusters, this bacterium should also acquire the ability to control polysaccharides biosynthesis in order to migrate toward the eutrophic zone and photosynthesize. In this evolutionary scenario, two possibilities could be considered (Fig. S8). First, this bacterium may have acquired both PGC and the *embRS* operon concomitantly from a photosynthetic α-proteobacterium. In this sense, it is intriguing enough that the *embRS* operon is immediately located downstream the PGC in *R. gelatinosus*, which may indicate the transfer of a unique DNA fragment encompassing the PGC and the *embRS* operon. Second, this bacterium may have acquired the PGC and the *embRS* operon following two successive gene transfer events. In both cases, control of biofilm formation and exopolysaccharide production will allow the bacterium to move in the water column.

## Supporting Information

**Movie 1.** Cell aggregate of the ΔEmbRS mutant: a polymeric material surrounds the cells in the aggregate and serves as a physical barrier against the entry of the free cells in the polymeric aggregate. Transmitted light image was acquired after 2 h crystal violet stain. The movie represents 2 min of acquisition of the same culture.

**Movie 2.** Biofilm formation by the ΔEmbRS mutant. Formation of the bacterial veil around the toothpick scaffold. Cells start growing in the whole plate; then the shrinking of the biofilm occurs and stops when the veil comes in contact with the top of the toothpicks, generating an organized polygonal network (Fig. 3).

**Figure S1.** Autoaggregation ability of the wild type and the ΔEmbRS null mutant. Cells were grown photosynthetically. The percentage of autoaggregation is expressed as: [(OD total- OD in suspension) ×100/OD total].

**Figure S2.** Crystal violet staining of ΔEmbRS cells form aggregate. The polymeric material delimited by * can be visualized in the movie M1.

**Figure S3.** Successive images of the biofilm formation around the toothpick scaffold. For more details see movie M2.

**Figure S4.** Examples of EmbRS-Tn5 mutants isolated in this study. (A) Mutants have recovered a partial or quasi-smooth phenotype compared to the parental ΔEmbRS null mutant on plates. (B) Photosynthetic liquid cultures of WT and ΔEmbRS compared with ΔEmbRS-GltA::Tn5, ΔEmbRS-Stik::Tn5 and to ΔEmbRS-BmfS::Tn5, transposon mutants.

**Figure S5.** (A) Organization of the genes encoding the BmfSR two-component system and the predicted domains within the histidine sensor kinase BmfS and the transcriptional regulator BmfR (Smart at EMBL). (B) Sequence alignment between EmbR and BmfR transcriptional regulators.

**Figure S6.** Putative regulatory model of the biofilm formation and autoaggregation in *Rubrivivax gelatinosus* based on the genetic and molecular data in this study. EmbRS functions as a negative two-component system that control genes (*cpsG, wcbM…*) involved in the synthesis of exopolysaccharides (extracellular matrix). BmfSR is a second two-component system also involved in biofilm formation. However, BmfSR should activate the expression of some genes involved in the synthesis of exopolysaccharides. The model includes also BdcA, a putative diguanylate cyclase, that may control exopolysaccharides and biofilm formation in *R. gelatinosus*.

**Figure S7.** Biofilm formation and sinking of the wild-type strain occur in the late stationary growth phase probably as a result of nutrient-limited environment. This event is accelerated in the ΔEmbRS mutant.

**Figure S8.** Putative role of EmbRS in the evolution of photosynthesis in *Rubrivivax gelatinosus*. Acquisition of *embRS* either concomitantly to the PGC (scenario 1) or after the PGC acquisition (scenario 2) may have allowed cells to migrate to the euphotic zone and to set up photosynthesis.

**Table S1.** Strains and plasmids.

**Table S2.** Primers.
