# Supplementary material for: EmbRS a new two-component system that inhibits biofilm formation and saves Rubrivivax gelatinosus from sinking
Source: Microbiologyopen. 2013 Mar 21;2(3):431–46. doi: 10.1002/mbo3.82 (PMC3684757; doi:10.1002/mbo3.82)
Supplement: Supplementary file 3 [file mbo30002-0431-SD3.pdf]

Supporting Information

Videos

Movie 1. Cell aggregate of the ΔEmbRS mutant: A polymeric material surrounds the cells in the aggregate and serves as a physical barrier against the entry of the free cells in the polymeric aggregate. Transmitted light image was acquired after 2h crystal violet stain. The movie represents 2 minutes of acquisition of the same culture.

Movie 2. Biofilm formation by the ΔEmbRS mutant. Formation of the bacterial veil around the toothpick scaffold. Cells start growing in the whole plate; then the shrinking of the biofilm occurs and stops when the veil comes in contact with the top of the toothpicks, generating an organized polygonal network (Figure 3).
