# Supplementary material for: EmbRS a new two-component system that inhibits biofilm formation and saves Rubrivivax gelatinosus from sinking
Source: Microbiologyopen. 2013 Mar 21;2(3):431–46. doi: 10.1002/mbo3.82 (PMC3684757; doi:10.1002/mbo3.82)
Supplement: Supplementary file 4 [file mbo30002-0431-SD4.pdf]

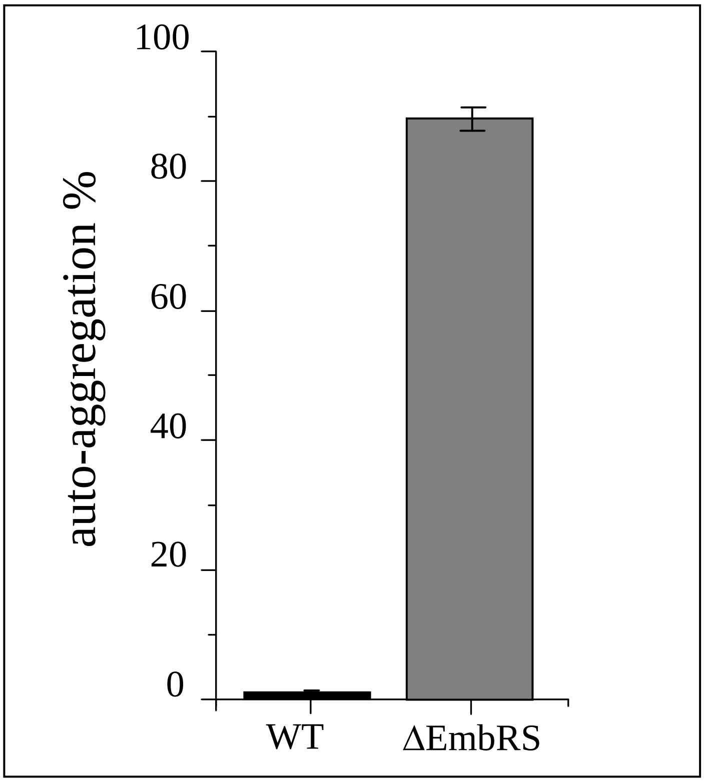

Figure S1. Auto-aggregation ability of the wild type and the ΔEmbRS null mutant. Cells were grown photosynthetically. The percentage of auto-aggregation is expressed as: [(OD total- OD in suspension) x100/ OD total].

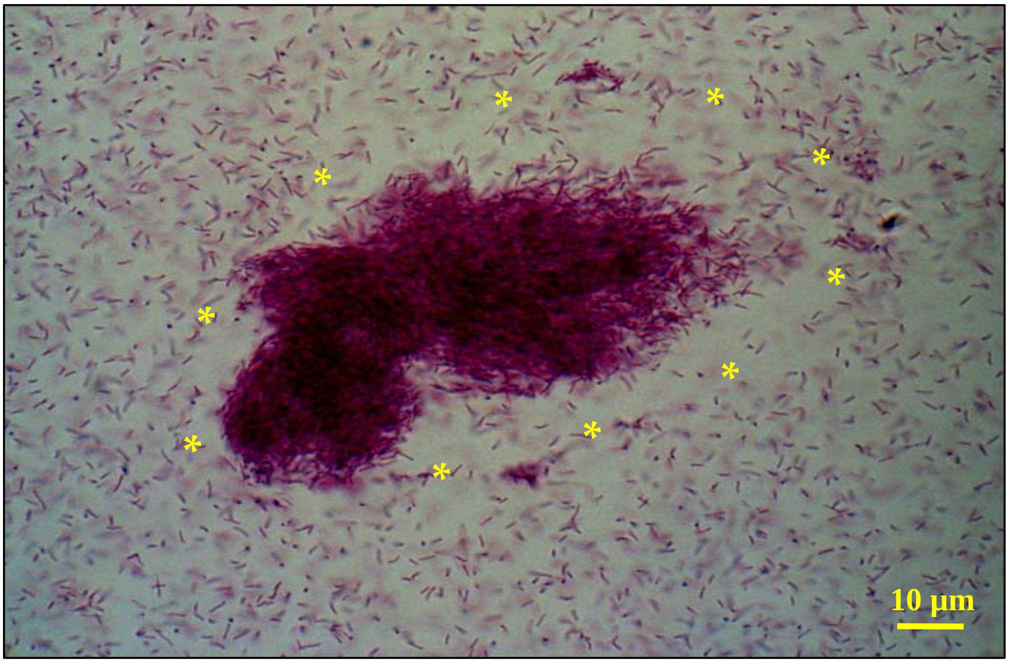

Figure S2. Crystal violet staining of ΔEmbRS cells form aggregate.
The polymeric material delimited by * can be visualized in the movie M1.

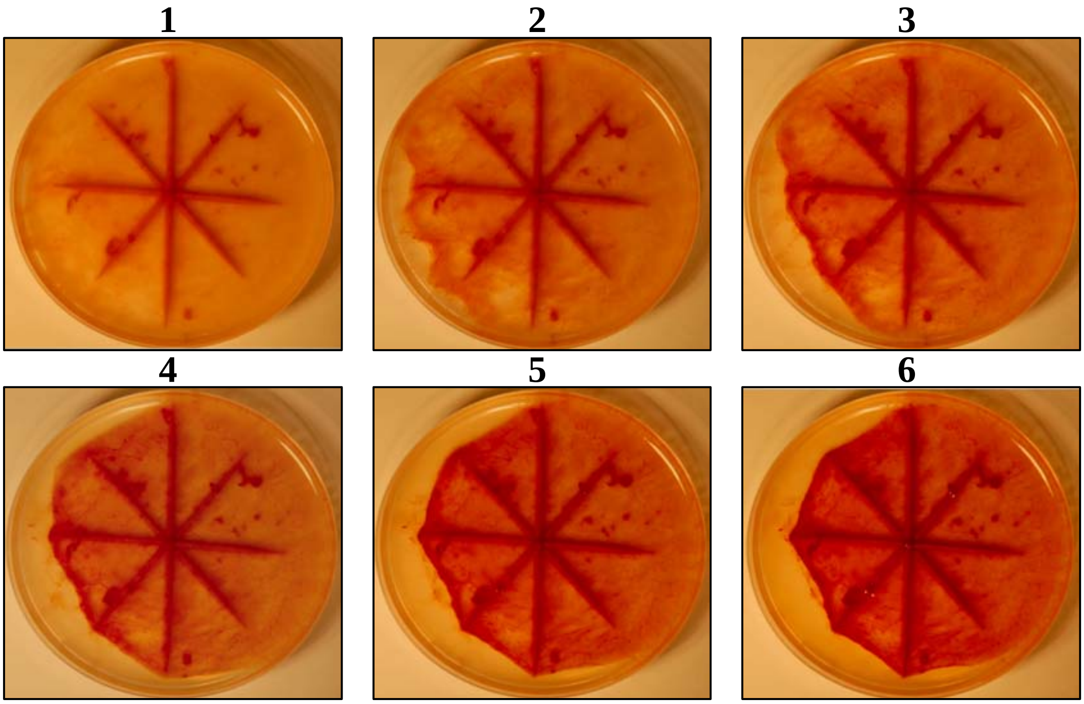

Figure S3. Successive images of the biofilm formation around the toothpick scaffold. For more details see movie M2.

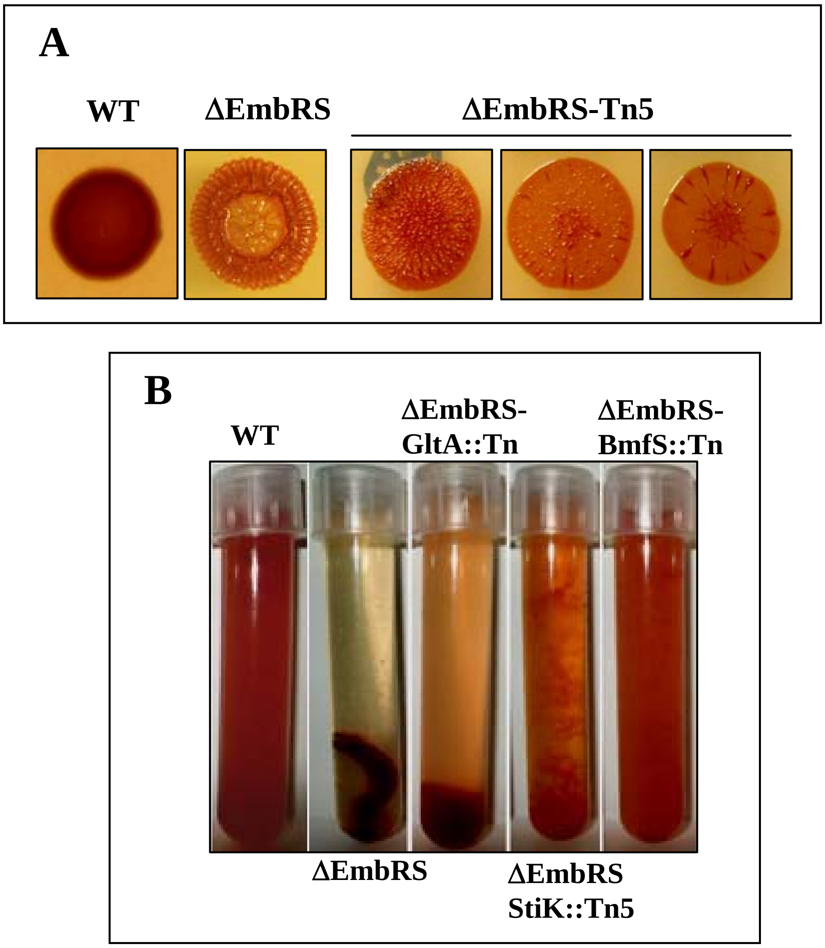

Figure S4. Examples of EmbRS-Tn5 mutants isolated in this study. **A**- Mutants have recovered a partial or quasi-smooth phenotype compared to the parental ΔEmbRS null mutant on plates.
B- Photosynthetic liquid cultures of WT and ΔEmbRS compared to ΔEmbRS-GltA::Tn5, ΔEmbRS-Stik::Tn5 and to ΔEmbRS-BmfS::Tn5, transposon mutants

A

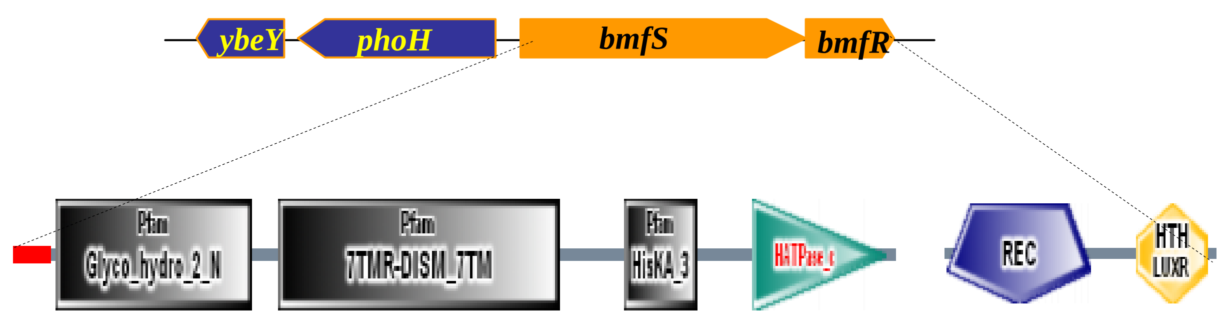

B

```
EmbR      MAVKVLILEDNPVARSFLCRVVRESFSDANHITEAGDLETARRHITLAGGATGLHGVDPF 60
BmfR      -MKNVLLLEDLPEIRAWMRKLVLQVFPTA-QISESARVQDA---ISLAAAVK-------F 48
             :**:*** *  *::: ::* : *. * :*:*:. :: *  *:**....       *

EmbR      KLILIDLELPDGNGMELLAELAHYP--ATKIVTTLYSDDEHLFPALQHGADGYLLKEDRF 118
BmfR      ELALIDLGLPDGSGVDVVTKLRDVQPDAQSVVVTIHDDDEHLFPALQAGAFGYILKEQPR 108
          :* **** ****.*:::::* .     * .:*.*::.********** ** **:***:

EmbR      EVLVEELQKIVRGQPPLSPAIARRLLTHFRHGAGPDVAPDSGFVNTTGFTTSRPVPMEKA 178
BmfR      ELIVEQLQRISQGEPPLSPSIARRMMAHFTQKAKPQTS---------------------- 146
          *::**:**:* :*:*****:****:::** : * *::
                                                        Δ

EmbR      LPEHERLTPRESEVLTYLSKGFTIKEIASLMGIKWFTVNDHIKSIYKKLNVSSRAEAAVL 238
BmfR      LLPHVQLTDRESEVLLRVAKGYTLPEIGVQLGLSRHTIADYVKQIYRKLNVSSRAEAALE 206
          *  * :** ******  ::**:*: **.  :*:. .*: *::*.**:***********:

EmbR     ASKQGLV- 245
BmfR     AQRLGLFR 214
         *.: **.
```

Figure S5. A- Organisation of the genes encoding the BmfSR two component system and the predicted domains within the histidine sensor kinase BmfS and the transcriptional regulator BmfR (Smart at EMBL). **B-** Sequence alignment between EmbR and BmfR transcriptional regulators.

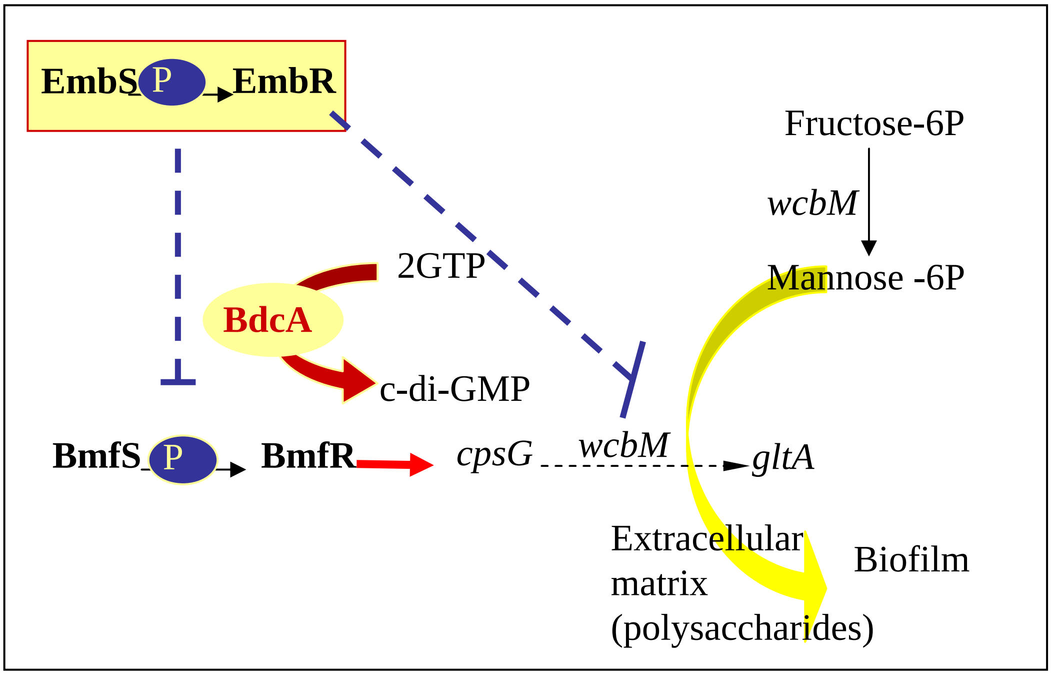

Figure S6. Putative regulatory model of the biofilm formation and auto-aggregation in *R. gelatinosus* based on the genetic and molecular data in this study.
EmbRS functions as a negative two component system that control genes (*cpsG*, *wcbM* …) involved in the synthesis of exopolysaccharides (extracellular matrix).
BmfSR is a second two component system also involved in biofilm formation. However, BmfSR should activate the expression of some genes involved in the synthesis of exopolysaccharides.
The model includes also BdcA, a putative diguanylate cyclase, that may control exopolysaccharides and biofilm formation in *R. gelatinosus*.

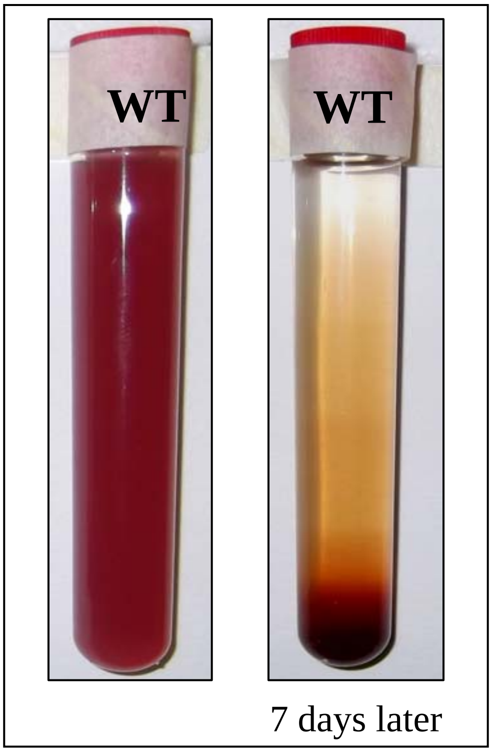

Figure S7: Biofilm formation and sinking in the wild type strain occur in the late stationary growth phase probably as a result of nutrient-limited environment. This event is accelerated in the ΔEmbRS mutant.

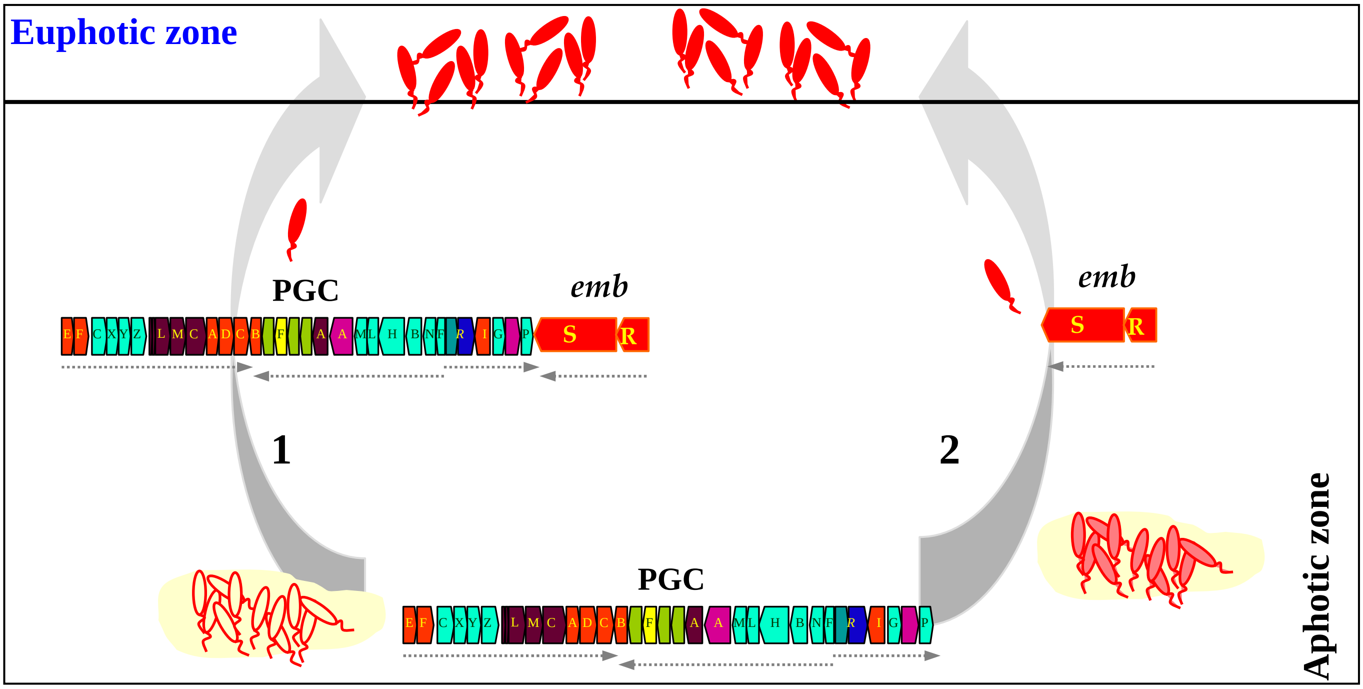

Figure S8. Putative role of EmbRS in the evolution of photosynthesis in *R. gelatinosus*. Acquisition of *embRS* either concomitantly to the PGC (scenario 1) or after the PGC acquisition (scenario 2) may have allowed cells to migrate to the euphotic zone and to set up photosynthesis.
