# Supplementary material for: EmbRS a new two-component system that inhibits biofilm formation and saves Rubrivivax gelatinosus from sinking
Source: Microbiologyopen. 2013 Mar 21;2(3):431–46. doi: 10.1002/mbo3.82 (PMC3684757; doi:10.1002/mbo3.82)
Supplement: Supplementary file 5 [file mbo30002-0431-SD5.pdf]

Table S1: Strains and Plasmids

Strains or plasmids	Relevant characteristics	Source
**Strains**		
E. coli		
JM109	F′ *traD36 proA*$^+$*B*$^+$ *lacI*^{q} *Δ(lacZ)M15/ Δ(lac-proAB) gln4 e14*$^-$ *gyrA96 recA1 relA1 endA1 thi hsdR17*	Promega
DH5α	F$^-$ endA1 gln4 thi-1 recA1 relA1 gyrA96 deoR nupG Φ80d*lacZ*ΔM15 Δ(*lacZYA-argF*)U169, hsdR17(r_K^- m_K^+), λ^-	Invitrogen
R. gelatinosus		
Strain S1	Wild type	(1)
EmbSΩ	*embS* disrupted strain (*embS*::Ω)	This work
EmbRK	*embR* disrupted strain (*embR*::Km)	This work
ΔEmbRS	*embR* and *embS* deleted strain (Δ*embRS*::Ω)	This work
ΔBmfR	*bmfR* deleted strain (*bmfR*::Tp)	This work
StiK	*Stik* disrupted strain (*stik*::Tp)	This work
ΔEmbRS-BmfRTp	*embR*, *embS* and *bmfR* deleted strain (Δ*embRS*::Ω- *bmfR*::Tp)	This work
ΔEmbRS-StiKTp	*embR*, *embS* and *stiK* deleted strain (Δ*embRS*::Ω- *stik*::Tp)	This work
**Plasmids**		
pGEMT	cloning vector (Ap^r)	Promega
pDrive	cloning vector (Ap^r Km^r)	Qiagen
pKS	pBluescript KS$^+$, cloning vector (Ap^r)	Promega
pUC4K	plasmid bearing the Km cartridge (Ap^r Km^r)	Pharmacia
pDW9	plasmid bearing the Ω cartridge (Ap^r Sp^r Sm^r)	(2)
p34S-Tp	plasmid bearing the Tp cartridge (Ap^r Tp^r)	(3)
pBBRMCS-3	Expression vector with tetracycline resistance	(4)
pSMS	pGEMT+ 2.15 kb *embS* fragment obtained with the primers ol-468 and ol-459	This work
pSMSR	pGEMT+ 2.6 kb *embSR* fragment obtained with the primers ol-489 and ol-475	This work
pSMSΩ	Ω cartridge inserted in the XmaI site within *embS* from pSMS	This work
pSMSRK	Km cartridge inserted in the EcoNI site within *embR* from pSMSR	This work
pSMΔΩ	Ω cartridge inserted in the AfeI site within *embR* and *embS* from pSMSR	This work
pDEmbRS	pDrive + 3.5 kb *embRS* fragment obtained with primers 159-160	This work
pDEmbR	pDrive + 1.3 kb *embR* fragment obtained with primers 158-160	This work
pDStik	pDrive + 2 kb *stiK* fragment obtained with primers 156-157	This work
pKStik	KS + XbaI/KpnI 2 kb *stiK* fragment isolated from pDStik	This work
pKStikTp	Tp cartridge inserted in the EcoRV site within *stiK* from pKStik	This work
pDbdcA	pDrive + 2 kb *bdcA* fragment obtained with primers 154-155	This work
pDbmfR	pDrive + 1.4 bmfR fragment obtained with primers 125-126	This work
pDbmfRTp	Tp cartridge inserted in the BglII sites within *bmfR* from pDbmfR	This work
pBembRS	pBBR1MSC3 + XbaI/KpnI 3.5 kb *embRS* fragment isolated from pDEmbRS	This work
pBembR	pBBR1MSC3 + XbaI/KpnI 1.3 kb *embR* fragment isolated from pDEmbR	This work
pBStik	pBBR1MSC3 + XbaI/KpnI 2 kb *stiK* fragment isolated from pKStik	This work
pBbdcA	pBBR1MSC3 + XbaI/KpnI 2 kb *bdcA* fragment isolated from pDbdcA	This work
pBbmfR	pBBR1MSC3 + XbaI/KpnI 1.4 kb *bmfR* fragment isolated from pDbmfR	This work

Ap^r, ampicillin resistant, Km^r, kanamycin resistant, Tp^r, trimethoprim resistant, Tc^r, tetracyclin resistant, Ω, Sp^r, Sm^r, spectinomycin and streptomycin resistant.

1. Uffen, R. L. (1976) *Proc. Natl. Acad. Sci. USA* **73**, 3298-3302
2. Prentki, P., and Krisch, H. M. (1984) *Gene* **29**, 303-313
3. Dennis, J. J., and Zylstra, G. J. (1998) *Appl Environ Microbiol* **64**, 2710-2715
4. Kovach, M. E., Phillips, R. W., Elzer, P. H., Roop II, R. M., and Peterson, K. M. (1994) *BioTechniques* **16**, 800-802
