# Supplementary material for: EmbRS a new two-component system that inhibits biofilm formation and saves Rubrivivax gelatinosus from sinking
Source: Microbiologyopen. 2013 Mar 21;2(3):431–46. doi: 10.1002/mbo3.82 (PMC3684757; doi:10.1002/mbo3.82)
Supplement: Supplementary file 6 [file mbo30002-0431-SD6.pdf]

Table S2: Primers.

Genes	Primers	Sequence
embR	Ol-489	GCGCCCGCCCCTGCCCTTCGT
	Ol-475	GGGGTTGGCCTCGATGCGGTAG
	RG158_embRF	GCTACCTGGTCGTCGGTTT
	RG160_embRR	AGGTCGTCAGCACGGAAG
embS	RG161_embSF	TCAACGACCACATCAAGTCC
	RG159_embSR	GACTTCGTCGCCAACCTCT
	Ol-468	GCTGACGCACTTCCGCCACG
	Ol-459	AGCGTGTGCCGGATGTAG
stiK	RG156_StiKF	GCCGCAAAACAGGATCATAG
	RG157_StiKR	GGTCTCAGCCACGGAACAGT
bdcA	RG154_bdcAF	GTCTCCCATGACCCTGGAT
	RG155_bdcAR	AGTCGGCGATCGACTTCTAC
bmfR	RG125_bmfRF	CGACGAGCAACATCATCAAG
	RG126_bmfRR	GTGAGAAAGCCGAAGAGCTG
gltA	RG123_glT-RTF	TCGTAATGCCGTGCTACAAC
	RG124_glT-RTR	GCCGCCATGATGAATATACC
bmfR	RG127_bmfRRTF	ATGAAGAACGTCCTGCTGCT
	RG128_bmfRRTR	ACGTTGAGCTTGCGGTAGAT
wcbM	RG129_wcbMRTF	GTTCGAACTCGCGGTGCT
	RG130_wcbMRTR	CCGATGTCGATGAACAGACC
cpsG	RG131_cpsGRTF	CAAGACGATCGACGAGACCT
	RG132_cpsGRTR	CCTTGCTGTACAGGTCGATG
pucB	Ol-539	GGCAGATGATGAAACAAG
	Ol-540	GTGGAGCCACGGCGTATAG
embR	RG168_embRF	AGCTGATCCTCATCGACCTG
	RG169_embRR	AAGCCCTTGCTGAGGTAGGT
